# Reviewers in 2016

**DOI:** 10.1098/rsos.170299

**Published:** 2017-05-10

**Authors:** Jeremy K. M. Sanders

**Affiliations:** Editor-in-Chief

Since the journal launched in 2014, the Editors of *Royal Society Open Science* have been exploring ways to recognize the hard work that peer reviewers contribute to published manuscripts. We have partnered with the peer reviewer recognition service Publons (https://publons.com/journal/23451/royal-society-open-science) to give referees the opportunity of claiming credit for their work.

In response to a recent survey of referees, who overwhelmingly indicated that they would welcome greater public recognition of their efforts by the journal, *Royal Society Open Science* and the Editors would like to thank all reviewers who have worked on papers published in 2016.

In common with our sister journal *Biology Letters*, to provide an opportunity for recognition, we list the names of all reviewers (unless they have opted out). This article is made permanent and citeable by its digital object identifier (DOI). We encourage you to quote your contribution in your applications for tenure and grants or other forms of research assessment. Where you have provided it, we have included your ORCID so your contribution can be unambiguously assigned to you.

Abd-Alla AM

Abdelaziz M

Abraham P

Abrams M

Adami C

Adams B

Adnet S

Afferrante L

Agardy T

Aglioti SM

Aguirre W

Ahlberg P

Ahnesjö I

Akcay E http://orcid.org/0000-0001-8149-7124

Albuquerque N

Aldous D

Allen B http://orcid.org/0000-0002-9746-7613

Allen P

Allen V

Allentoft M

Allison GT

Almirantis Y

Alvarado S

Amack J http://orcid.org/0000-0002-5465-9754

Amano M

Amici F

Aminikhah H

Anantharaman K

Andersson L

Andersson M

André J-B

Andrews P

Andris C

Angelsky O

Annunziata O

Anthes N

Antonietti M

Aoki K

Arcese P

Archie E

Arim M http://orcid.org/0000-0002-7648-8909

Armitage S http://orcid.org/0000-0002-5561-9543

Arnould O

Arntzen J

Asami T

Atangana A

Atkins A

Attasart P

Attenborough R

Aureli F

Austin J http://orcid.org/0000-0003-4244-2942

Ayasse M

Aykanat T

Ayling J

Azeloglu E http://orcid.org/0000-0001-6137-109X

Baber C

Bacchetti P

Bachrach A

Bajaj-Elliott M

Baker D http://orcid.org/0000-0002-0161-443X

Baldwin A

Baldwin M

Baleanu D

Balmer B

Banerjee N

Banissy M

Bank C

Banks C

Bañuelos J

Barber I

Bard K

Barker D

Barluenga M

Barnett A

Barons M

Barreiro A

Barreto E

Barrett L

Barrientos A

Barta Z

Barthelemy M

Barton L

Bartos M

Barve S http://orcid.org/0000-0001-5840-8023

Basari N

Batalha-Filho H

Bates M

Batty C

Becher M

Bednekoff P

Beguerisse Diaz M http://orcid.org/0000-0002-8750-8346

Behar M

Belk M

Bell H

Bell M

Bellomo N

Bellstedt D

Benadi G

Bendrey R

Bennett S

Bennett W

Benoit-Bird K

Berbesque C

Berryman M

Berta A

Bertora F

Best J

Bex P

Bhadra A http://orcid.org/0000-0002-3717-9732

Bhat A http://orcid.org/0000-0002-7447-2380

Birch D

Biro D

Bischoff M

Bitton P-P http://orcid.org/0000-0001-5984-2331

Blaauw B

Blanke A http://orcid.org/0000-0003-4385-6039

Bleak C

Block P

Blunt LA

Blythe R

Bobak A

Boeger W

Bogacz R

Bohbot J

Bohmann K

Bok M

Bokes P

Bonebrake T

Bonisoli-Alquati A

Bonnie K

Booth D

Borah D

Bothwell M

Bouffanais R

Boulter L

Bowman D http://orcid.org/0000-0001-8075-124X

Boyd C

Bradley A

Braga Goncalves I

Braswel G

Braun EJ

Brazil I

Breed G

Brelsford C

Brembs B

Brenner E

Brewer R http://orcid.org/0000-0001-8170-7590

Brinkløv S

Brinkman D

Briscoe J

Britton T

Brodin A

Brosnan S http://orcid.org/0000-0002-5117-6706

Brown C

Brown K

Brown R

Brownbill P

Bru A

Bruijn S

Brule T

Brumm H http://orcid.org/0000-0002-6915-9357

Bshary R

Budd G

Budischak S

Bulmer M

Bunin G

Bunyak F

Burda H

Burr D

Burroughs R

Burrows A

Bustnes JO

Byrne M

Cabreiro F

Cade D

Cai J

Calò A

Camargo P

Campbell C

Campbell M

Canals M

Candidi M

Canner J

Canteloup C

Cantor M http://orcid.org/0000-0002-0019-5106

Capraro V

Carbon C-C

Cardé R

Cardoso G http://orcid.org/0000-0001-6258-1881

Caro T http://orcid.org/0000-0001-6804-8519

Carpentieri G

Carranza S

Carter C

Carter G http://orcid.org/0000-0001-6933-5501

Cartwright R

Caruso N

Casas J

Casasanto D

Cass J

Castellote M

Cavallo L

Cavanaugh C

Ceacero F http://orcid.org/0000-0001-9014-147X

Celeux G

Chakrabarty D

Challis J

Chapple K

Chapple T

Chapuisat M http://orcid.org/0000-0001-7207-199X

Charlton N

Charness G

Chase B

Chassin-Noria O

Chatterjee A

Chatterjee S

Chauvaud L

Chaverri G

Chen G http://orcid.org/0000-0003-1381-7418

Chen W

Chen X

Chenchiah I

Cheung WWL

Chiao C-C http://orcid.org/0000-0001-9506-0230

Chiao J-C

Chisholm J

Chivers M

Chockalingam N

Chousiadas K

Chowell G http://orcid.org/0000-0003-2194-2251

Christy J

Cicchini GM

Cillessen A

Cirulli E

Claessens L

Clairambault J

Claret L

Clark A http://orcid.org/0000-0001-5908-2862

Clarke A

Cleeves LI

Clements C

Clements J http://orcid.org/0000-0001-5140-5751

Clomburg J

Cochran J

Cockcroft P

Codd J http://orcid.org/0000-0003-0211-1786

Cohen A

Coleman R

Coltman D

Combes S

Condamine F

Conway J

Coombe D

Coomes D

Cooper S

Corcoran K

Cordonnier T

Corlett R

Cormier S

Cornuault P-H

Correll N

Corucci F

Coscia M

Cosentino C

Costa D

Costa L

Costalago D

Cowan N

Cownden D

Cox M

Cramp R

Creath R http://orcid.org/0000-0002-7522-7192

Cresti M

Cristol D

Croft D

Cross F http://orcid.org/0000-0001-8266-4270

Cuevas-Yanez E

Cunningham S

Currie C

Cuthill I

Cvekl A

Czaczkes T http://orcid.org/0000-0002-1350-4975

Daamen W

Daesslé L

D'Alba L

Dale J

Dall S http://orcid.org/0000-0001-9873-6507

Dall'Asta L

Dana J

Danforth B

Dang C

Danks D

Darch S

Darimont C http://orcid.org/0000-0002-2799-6894

Darvishi S

Das J

David C

Davies S http://orcid.org/0000-0003-3890-7372

Davis K

Dawes J

Dawson R

de Goeij J

de Gosson M

de la Parra R

De Lisle S

de vries F

DeAngelis DL

Dececchi A

Dechaume-Moncharmont F-X

Dehling M

deKat R

Dell'Olio F

DeLong J

Delton A

Demarest A

Demeritt D

DeMiguel D

Derkach S

Dermon C

Derrible S

Desai T

Dewhirst O

Di Molfetta G

Di Santo V

Dickens J

Dickinson G

Dickman C

Dienes Z

Dierssen H

Dingwell J

Dinis L

Dinwiddie A

Dixson B

Dixson D

Domenici P http://orcid.org/0000-0003-3182-2579

Dommers S

Doncaster C

Dong X

Donner R

Donovan S

Dorogovtsev S

Dorr M http://orcid.org/0000-0002-7879-7908

Douhard M http://orcid.org/0000-0001-9422-7270

Drake A

Drake A

Drasdo D

Du D

Duarte Queirós S

Dubuc C http://orcid.org/0000-0001-8855-2923

Duclot F

Duda P

Dudley R

Dunbar R http://orcid.org/0000-0002-9982-9702

Duncan M

Dunham Y

Dunn R

Dunn S-J

Dupont J

Duque J

Durban J

During H

Durrant S

Durrett R

Dyke G

Ebbe B

Ebensperger L

Eder A

Edwar C

Edward B

Edwards D http://orcid.org/0000-0001-8562-3853

Edwards P

Eisenegger C

Eisert R

Elgendi M

Elias D

Elkin B

Ellis P

Ellsworth R

Enders E

Endler J http://orcid.org/0000-0002-7557-7627

Engelhard GH

Engelhardt A

Eppell S

Epskamp S

Ercit K

Erdmann T

Erler S

Escobedo-Morales L

Escobedomx C

Etessami K

Etheridge A

Etter R

Evans J http://orcid.org/0000-0002-2603-6832

Eyre-Walker A

Ezcurra M

Faltings B

Farine D http://orcid.org/0000-0003-2208-7613

Farkas I

Farkas T

Farke A

Farmer C

Farmer H

Farrell R

Farrell T

Farrell W

Farris D http://orcid.org/0000-0002-6720-1961

Fauve S

Fearn H

Fearnhead P

Felson M

Feng N

Feng X-Q

Fenton B

Fernandez C

Fernhoff N

Ferrari M http://orcid.org/0000-0002-3127-9804

Ferruzzi M

Festa-Bianchet M

Fey N

Fiedler W

Field D

Filingeri D http://orcid.org/0000-0001-5652-395X

Fischer J

Fischer S

Fisher M http://orcid.org/0000-0002-1862-6402

Fitzgerald S

Flagel D

Flaxman S

Flaxman S

Fleischer R

Fletcher J

Flitney A

Flower C

Flower T http://orcid.org/0000-0003-0531-8010

Fogarty L

Forasiepi A

Fordyce RE

Fornai C

Forney K

Förster S

Foth C http://orcid.org/0000-0002-9410-4569

Foulkes L http://orcid.org/0000-0002-8122-4270

Fozard J http://orcid.org/0000-0001-9181-8083

Frank S

Frankenhuis W

Franks N

Franssen N

Frantz L

Frasch W

Fratkin J

Frederiksen M

Freeman M

Frey ED

Friedlingstein P

Friedman M

Friedrich M

Friedrich M

Fu H

Fujita K

Furlong P

Gabriele C

Galati A

Galtier N

Gamble A

Gangestad S

Ganias K

Ganopolski A

Gao H

Gao Y

Gao Z-K

Garcia-Palacios A

Garder PE

Garland E

Garnham A

Garnier R

Garra R

Garthe S

Garthwaite J

Garwood R

Gattas J

Gaume L

Gautron J

Gawande MB

Gebauer G

Gebo D

Gehringer M

Geiser F

Genkai-Kato M

Georges A

Georgiev A

Getzin S

Geyer H

Ghanbarnejad F

Giani L

Giannini TC

Gibbs J

Gibson B

Gibson M

Gidoin C

Gienapp P

Gilbert C

Gilbert J

Gilbey J

Gillam E

Gillis A

Gingins S

Ginzberg P

Giordano M

Giroud S

Glatzel M

Gleason T

Gneezy A

Godwin J

Goetz K

Goka K

Goller F

Gómez-Liévano A

Gonçalves B

Gonder MK

Gonzalez-Tokman D

Goodacre S

Goodchild M

Gopalakrishman S

Gordon D http://orcid.org/0000-0002-1090-9539

Gortazar C

Gowans S

Goymann W http://orcid.org/0000-0002-7553-5910

Grabowski D

Graciá Martínez E

Grandi L

Grant R

Gray D http://orcid.org/0000-0002-4439-561X

Gray R

Grebenkov D

Grebogi C

Gredeback G

Greenwood J

Greenwood P

Gremillion K

Griffin A

Griffith M

Griffiths R

Grima R http://orcid.org/0000-0002-1266-8169

Gronlund S

Groothuis T

Groundwater PW

Gruber T

Gu L

Guberti V

Guillen N

Guimaraes R http://orcid.org/0000-0002-6947-9967

Gumm J

Gunnell G

Güntürkün O

Gurney J

Hafner M

Hagedorn M

Halfwerk W

Hall G

Halloy J http://orcid.org/0000-0003-1555-2484

Hall-Spencer J

Halsey L http://orcid.org/0000-0002-0786-7585

Hamby K

Hancock P

Hansell M

Hansen A

Hanus R

Happer W

Hardwicke T http://orcid.org/0000-0001-9485-4952

Harfoot M

Harkrider A

Harper L

Harris J http://orcid.org/0000-0002-3497-4503

Harrison W

Harrod C http://orcid.org/0000-0002-5353-1556

Harvey B http://orcid.org/0000-0002-4971-1634

Hasegawa T

Hasegawa U

Haselgrove M

Hasell T

Hatano N

Hatchwell B

Hauber M

Hauquier F

Hauser C

Hauser O

Havlíček J

Hayden B

Hearn A

Heffernan J

Heimbauer L

Heintz M

Heintzelman S

Helanterä H

Helle S

Heller R

Hellweger F

Hembry D http://orcid.org/0000-0002-4907-8912

Henderson T http://orcid.org/0000-0002-5028-9047

Henningsson P

Henriques R http://orcid.org/0000-0002-6544-5532

Hensley B

Henson F

Henzi P

Herbeck J

Herbert-Read J http://orcid.org/0000-0003-0243-4518

Herrel A

Hessen D

Heubel K http://orcid.org/0000-0002-7946-5542

Higgins J

Higginson A http://orcid.org/0000-0002-2530-0793

Hincke M

Hino H

Ho J

Hobson E

Hoekstra J

Hoelzel AR

Hoey A

Hoffman P

Hoffmann F

Hoffmann M

Hofreiter M http://orcid.org/0000-0003-0441-4705

Holliger P

Holowka N

Holroyd P

Holt N

Holveck M-J

Hone D

Hopcroft P http://orcid.org/0000-0003-3694-9181

Horn A

Horton T

Hoshino T

Hoskisson P

Hotchkin C

Hu H

Hu L-q

Hu X

Hu X

Hua X

Huang J

Hubel T http://orcid.org/0000-0002-6749-6747

Hubenova J

Hubicki C

Huchard E

Hueckel T

Hughes A http://orcid.org/0000-0003-2677-1965

Hulsey CD http://orcid.org/0000-0002-9653-6728

Humphries N

Humston R

Hunter L

Huntley B

Huntley J

Hussein B

Huster D

Hutson K

Hwang N

Hyman J

Igic B http://orcid.org/0000-0002-3219-6381

Iguchi A

Iida F

Ikeda Y

Imbert E

Inouye D

Inzoli F

Ioannou S

Ion R-M

Iosilevskii G http://orcid.org/0000-0002-4114-3214

Iserbyt A

Ispolatov I

Ivlev A

Ivory J

Jackson G

Jakob E

Jakobsen ML

Jalil N-E

James H

Jandzik D

Jansson F

Janz D

Jeanniard du Dot T

Jeffery K

Jensvold ML

Johnson K

Jolles J http://orcid.org/0000-0001-9905-2633

Jones A

Jones P

Jordal B

Jordan A

Jordan F

Jordan J

Joseph L

Josserand C

Joufflineau C

Joyce W

Joyner-Matos J

Ju F

Jung J-L

Jurčišin M

Jurdak R

Kaburu S http://orcid.org/0000-0001-7456-3269

Kagawa N

Kamel S

Kamerlin SCL http://orcid.org/0000-0002-3190-1173

Kandler A

Kantelhardt J

Karamanlidis A

Karamanos N

Kardos M

Karshafian R

Kasuya T

Kautz M

Keagy J

Keeling L

Keijsers N

Kelber A http://orcid.org/0000-0003-3937-2808

Kellar N

Kellner A

Kello C

Kelly C

Kelly D

Kemp J

Kempenaers B

Kennedy A

Kepaptsoglou K

Kerkhof AJ

Khanna V http://orcid.org/0000-0002-7211-5195

Kievit R

Kilner J

Kilner R

Kimmel R

King L

Kishi N

Kjellberg F

Kjesbu O

Klages R

Klaren P

Klump B

Knapp R

Knell R

Kobayashi M

Kocifaj M

Koeppl T

Kondo N

Konijnendijk CC

Konnerth J

Korosi J

Korth M

Kothe D

Kotrschal A http://orcid.org/0000-0003-3473-1402

Koyama N

Krajewski C

Kranioti E

Krasileva K

Kreiman J

KRISHAN V

Krishnan S

Krist M

Krützen M

Kühlbrandt W

Kukekova A

Kuroe Y

Kutsukake N

Kwak Y-S

Lacarbonara W http://orcid.org/0000-0002-8780-281X

Lacasa L

Lakens D

Lal K

Lam F-P

Lambert J

Lammers M

Langman V

Laschi C

Lattin C http://orcid.org/0000-0003-4030-4212

Latzman R

Laughton A

Lautner G

Lawrence D

Lea S

Leavens D

Leaver L

Lebrasseur O

Lee A

Lee D

Lee J

Legalov A

Lehikoinen A

Lehmann J

Lehtonen T

Leonard J

Leonards U

Lepora N

Lesku J

Lesser M

Levin L http://orcid.org/0000-0002-2858-8622

Levin S

Levnajic Z

Lewis R

Li C

Li G

Li L

Li Z

Liao Q

Lichtwark G

Lima-Mendez G

Lin C-H

Lin W http://orcid.org/0000-0002-1863-4306

Lind O http://orcid.org/0000-0002-5490-4705

Linding R

Lindstrom J

Lindström K

Linhart P

Linko V

Lipke E

Lisney T

Liu G

Liu PP

Liu X

Lloyd D

Logan C http://orcid.org/0000-0002-5944-906X

Logares R

Lord K

Loske A

Louf R

Lovejoy T

Lu G

Lu Y http://orcid.org/0000-0002-9280-2718

Lu Z

Lucia U http://orcid.org/0000-0002-3123-2133

Luck C

Lulli G

Lundkvist P

Lv X

Lythgoe K

Ma X

MacDonald B

MacDougall A

Mace R http://orcid.org/0000-0002-6137-7739

Machovsky-Capuska G

Macías-García C

Mackenzie S

Macleod M

Madigan D

Maibach E

Majolo B http://orcid.org/0000-0002-0235-3040

Maldonado J

Mallela K

Manem V

Manley E

Mann J

Mannion P

Mansmann U

Marchiori P

Marden J

Marinazzo D

Marsh H

Marsh R

Marshall J

Martén-Rodríguez S

Martin A

Martin S

Martin-Duran J-M http://orcid.org/0000-0002-2572-1061

Martini X

Mason A

Mastrandrea R

Masuda N http://orcid.org/0000-0003-1567-801X

Masuda T

Masunov A http://orcid.org/0000-0003-4924-3380

Matisoo-Smith L

Mattea C

Matthews L http://orcid.org/0000-0001-7889-5228

Mattys S

Matz M

Maurer G

Maximino C

Maxson R

May K

May ML

McAuliffe K

Mcdonald-Madden E

McGhee K

McGuire L

McKee S

McKelvey K

McKey D

McNaughter P http://orcid.org/0000-0002-9330-389X

McNeely C

McWethy D

Mehlis M

Meldon J

Melloni L

Melson G

Mench J

Mendelson T

Méndez V

Mennill D http://orcid.org/0000-0001-9314-6700

Mensink P

Menzel CR

Merilä J

Mesoudi A http://orcid.org/0000-0002-7740-1625

Meynet G

Miatto F

Michel F

Michoel T

Migliano A http://orcid.org/0000-0003-4364-2735

Miller III D

Miller C

Miller DS

Miller H

Miller J

Miller M

Mills G

Mills K

Milne N

Minamoto T

Minelli A

Minetti A

Mioduszewska B

Miotto J

Mirzaei B

Missier P

Miyashita S

Mjolsness ED

Mlynarek J

Molinero C

Møller A http://orcid.org/0000-0003-3739-4675

Molles L

Mondragon R

Monette M

Montfort W

Moran J

Moran R

Mori S

Morin C

Morin P

Morrison S

Moseby K

Moss R

Mouchet S http://orcid.org/0000-0001-6611-3794

Mougi A

Mourer-Chauviré C

Mousavi J

Moussaid M http://orcid.org/0000-0002-1268-6332

Moyà-Solà S

Mughal A http://orcid.org/0000-0002-1913-9382

Mukubana W

Mullally S

Muller E

Muller U

Müller W

Mullin C

Munch S

Murray-Rust D

Murry B

Murthy A

Müser M

Musolesi M

Muthukrishna M

Nagarajan R

Najafzadeh M

Nakabachi A

Nakagawa S

Nakashima S http://orcid.org/0000-0003-3992-8589

Nanayakkara C

Nanjundiah V

Naqavi I

Narayanan G

Natalini R

Natoli E

Natterer F

Neff B

Nelson D

Nelson F

Nelson W

Nerukh D

Neuberg S

Neuhaus B

Neve P

Newport R

Nezami FR

Nichols H http://orcid.org/0000-0002-4455-6065

Niedźwiedzki G

Nilsson G

Noë R

Nord C

Norenzayan A

Norros V

Nowack W

Nowak M

Nowell D

Nuño JC

Nuth J

Nyhan B

O'brien O

O'Donovan O

Ogden R

Ogilvie H

Ogura-Tsujita Y

Ohtsubo Y http://orcid.org/0000-0003-2074-0244

Ojanguren A

Ojima I

Okuyama J

Olsen MT

Olson D

Olsson L

Omachi H

O'Mara T

Orben R

Orchard S

Ortman S

Orton D

Osorio D

Ostojic L

Outram A

Ozdemir S

Padfield D

Pagès J

Paggi M

Paitz R

Palmer AR

Panagiotopoulou O http://orcid.org/0000-0002-6457-448X

Panfilio K

Panksepp J

Pannala V

Papastamatiou Y http://orcid.org/0000-0002-6091-6841

Park A http://orcid.org/0000-0003-4080-7274

Parker B http://orcid.org/0000-0002-0679-4732

Parker L

Parkesh R

Parkinson J

Parry L http://orcid.org/0000-0002-3910-0346

Patil A

Patterson E

Pavei G

Pavey C

Payne N

Pearce P http://orcid.org/0000-0001-5788-3826

Pearce-Higgins J

Pearse D

Peart C http://orcid.org/0000-0002-0433-0299

Pedley T

Pekar S

Pelabon C

Pelz-Stelinski K

Peña E

Peña J

Peng Y-Q

Penner O

Penta R

Perc M http://orcid.org/0000-0002-3087-541X

Perez-Enciso M http://orcid.org/0000-0003-3524-995X

Perez-Lanuza G

Perna A

Petersen A http://orcid.org/0000-0002-0955-3483

Petit O

Petrovskii S http://orcid.org/0000-0001-6259-2695

Peysakhovich A

Piazza S

Pierce L

Pillon Y

Pin C

Plaza G

Podobnik B

Polin M

Pomiankowski A

Pond D

Pontzer H http://orcid.org/0000-0003-2397-6543

Poompuang S

Popov VL http://orcid.org/0000-0003-0506-3804

Poppinga S

Porfirio A

Potvin D http://orcid.org/0000-0001-9296-9297

Powles S

Pratt S

Pravosudov V

Primicerio M

Pröhl H

Prosenc M

Proudlock G

Prunet J

Ptasinska S

Puebla O http://orcid.org/0000-0001-9700-5841

Puritz J

Putman N http://orcid.org/0000-0001-8485-7455

Quattrociocchi W

Quinlan P

Quintana P

Quistberg A

Raap T

Radford C

Radhakrishnan S

Rafikov R

Rahbar N

RAMALHO A

Ramasco J

Ramcharan C

Ramirez-Lopez P

Ramstein G

Raorane M

Rašić G

Ratcliffe E

Raverty S

Rawlence N http://orcid.org/0000-0001-6968-6643

Rawlings B

Rayfield E

Read J http://orcid.org/0000-0002-9029-5185

Reddon A http://orcid.org/0000-0002-3193-0388

Redmond L

Redner S

Reiser P

Reitzel A

Ręk P http://orcid.org/0000-0001-5480-4053

Rekdahl M

Ren D http://orcid.org/0000-0001-8660-0901

Requier F

Restif O http://orcid.org/0000-0001-9158-853X

Reyes-Prieto A

Rhodes J

Ribeiro HV

Ribeiro RV

Rice L

Richter M

Ridgely R

Rieger G

Riffo V

Riley A

Riley N

Rime B

Ritson-Williams R

Roach N

Robertson B

Robillard T

Robinson K

Robinson M

Robinson-Rechavi M

Roche B

Rodrigues A

Rogers L

Rohner C http://orcid.org/0000-0001-8760-8972

Romero T

Romero-Barrios P

Rosenberg M

Rosengrave P

Rosenzweig M

Ross S

Rotkirch A http://orcid.org/0000-0002-9429-1499

Roulin A http://orcid.org/0000-0003-1940-6927

Rout T

Rowan J http://orcid.org/0000-0002-5062-4044

Rowe L

Rowley C

Roy S

Roy S

Ruban A http://orcid.org/0000-0001-8554-0249

Rubenson J

Rubenstein D http://orcid.org/0000-0002-4999-3723

Rubinov M

Rubio De Casas R http://orcid.org/0000-0003-4276-4968

Rubolini D

Rutkowska J

Ruuskanen S http://orcid.org/0000-0001-5582-9455

Rydell J http://orcid.org/0000-0002-4930-6110

Sacchi R

Sachell L

Sacks M http://orcid.org/0000-0002-2711-8370

Sagi D

Saiki Y

Saint D

Salo P

Sanei S

Sankar G

Santella A

Santos LND

Saroglou V

Save E

Sbalzarini I

Scarpino S

Schacht R http://orcid.org/0000-0002-4606-7345

Schaefer J

Schino G

Schläpfer M

Schlatter D

Schluter J

Schmidt M

schmidt-Rhaesa A

Schoener C

Scholz A

Schraagen JM

Schroeder J

Schwarz U http://orcid.org/0000-0003-1483-640X

Schwarzkopf D

Scott J

Scott R

Seebacher F http://orcid.org/0000-0002-2281-9311

Semple S

Sen A

Senff Ribeiro A

Sepil I

Servos P

Seyfarth R

Sferco E

Shackelford T

Shackell N

Shai S

Shankland M

Shanks D

Shatunov M

Shefelbine S

Shelton D

Shepard E

Sheppard P

Sherratt E

Shigenobu S

Shine R http://orcid.org/0000-0001-7529-5657

Shirai N

Shoji T

Shultz S http://orcid.org/0000-0002-7135-4880

Sierra E

Sievert S

Silk J

Silk M

Sills J

Simon R

Singarayer J

Sinton D

Sirois S

Skariyachan S

Sköld H

Skuk D

Slocombe K http://orcid.org/0000-0002-7310-1887

Smith B

Sobolevsky S

Sochi T

Solé A

Soler M http://orcid.org/0000-0002-6451-0793

Solomon J

Soniat T

Sonmezoglu A

South M

Sparks A

Spence A

Spence R

Staal A

Stack S

Stafford K

Steadman P

Steane D

Steel L

Štefka J

Steiger S

Steinruck H

Stevens M http://orcid.org/0000-0001-7768-3426

Stimpert A

Stockdale F

Stramaglia S

Streicker D

Stribling J

Stuber E

Sturdy C

Sugasawa S

Suhay E

Sullivan C

Sullivan M

Sun L

Sundin J

Sunnucks P

Suriyampola P

Sutton M

Svanback R

Swart A

Sword G

Syed T

Szekely T http://orcid.org/0000-0003-2093-0056

Szell M

Szolnoki A

Talhelm T

Tallmon D

Tambussi C

Tanskanen A

Tatsuya S

Taubert J http://orcid.org/0000-0002-6519-8068

Tavares J

Taverne L

Taylor C

Taylor G http://orcid.org/0000-0001-8289-755X

Temple S http://orcid.org/0000-0001-6447-4713

Tervo O

Thambynayagam M

Thibaut B

Thiran P

Thomas P

Thompson J

Thompson P

Thorne L

Thornhill D

Thuenken T

Thuy Ba Linh N

Tiunov A

Todd V

Todorov A

Toft S http://orcid.org/0000-0002-2839-3921

Togashi T http://orcid.org/0000-0002-9270-9863

Tomás G

Touchon J

Toussaint E

Townsend B

Trajanovski S

Tramontano A

Traxlmayr MW

Treiber M

Trémolière B

Tresguerres M http://orcid.org/0000-0002-7090-9266

Tressoldi P

Trimmer P http://orcid.org/0000-0003-0914-4150

Tropea C

Trujillo M

Tunney R

Turner T

Turoverov K

Tyler C

Tyminski J

Tzuk O

Urazghildiiev I

Vaglio S

Valkonen J

Vallortigara G

van Belzen J

van Duijn M

van Elk M

van Gennip Y

van Oort N

van Zweden J

Vanaverbeke J

Vanstreels R

Varshney L http://orcid.org/0000-0003-2798-5308

Vassiliev D

Vaudo J

Velasco D

Velez-Juarbe J

Velusamy V

Venable L

Vereecke E

Verones S

Vesper C

Vignier J

Vincent J

Vindas M http://orcid.org/0000-0002-3996-0952

Vinther J

Vlakveld W

Voelkl B http://orcid.org/0000-0001-5454-2508

Vøllestad L

Voloshina A

von Rintelen T

Vonk J

Vorobyev M

Vosoughi S

Wada H http://orcid.org/0000-0002-9594-3647

Wada K

Wagenmakers E-J

Wagner C

Wagner C

Waichert C

Wake M

Wakeling J

Walker T

Walling C

Walsh P

Walsh S

Walther B

Walton V

Wang L

Wang P

Wang Z

Ward A

Ward T

Ware J

Watanabe S

Watson I

Webb B

Weikl T

Weintraub P

Weir L

Weldon C

Wenzel M

Wepprich T

Westneat DF

White T

Whitenack L

Whitney B

Whitney D

Whittet D

Widmer L

Wiens J http://orcid.org/0000-0003-4243-1127

Wiertlewski M

Wikswo J

Wilkins M

Wilkinson F

Willführ K

Williams G

Williams H

Williams J

Wilson D

Wilson S http://orcid.org/0000-0002-4590-0948

Wilts B http://orcid.org/0000-0002-2727-7128

Wise S

Witmer L

Witt A

Witting L

Wixted JT

Wohlrabe K

Wolff E

Wolfram M-T http://orcid.org/0000-0003-1133-8253

Womble J

Wood C

Wood T

Wooten J

Wurm Y

Wursig B

Wyatt T http://orcid.org/0000-0003-0371-0713

Xiao D

Yamagata Y

Yamagiwa J

Yamamoto K

Yamawo A http://orcid.org/0000-0003-2928-8151

Yamazaki Y

Yan HY

Yan W

Yan X

Yang J

Yang J

Ye L

Yi W

Yopak K

Yoshimura J http://orcid.org/0000-0003-1610-1386

Yoshizawa M

Youn H

Zagalska-Neubauer M http://orcid.org/0000-0002-9738-1393

Zagrobelny M

Zakharov A

Zaltz Austwick M

Zamma K

Zamorano J

Závorka L

Zednik C

Zhan X

Zhang B

Zhang H

Zhang Q

Zhang Z

Zhu Y

Zietriz A

Zomorrodi A

Zubarev R

Zuest T

Zuk M

